# Targeting Aggressive Cancer Stem Cells in Glioblastoma

**DOI:** 10.3389/fonc.2015.00159

**Published:** 2015-07-20

**Authors:** Tracy Seymour, Anna Nowak, Foteini Kakulas

**Affiliations:** ^1^School of Medicine and Pharmacology, Faculty of Medicine, Dentistry and Health Sciences, The University of Western Australia, Crawley, WA, Australia; ^2^Hartmann Human Lactation Research Group, School of Chemistry and Biochemistry, Faculty of Science, The University of Western Australia, Crawley, WA, Australia

**Keywords:** glioblastoma, gliosarcoma, octamer-binding transcription factor 4, SRY (sex determining region Y)-box 2, Nanog homeobox, pluripotency genes, glioma stem cells

## Abstract

Glioblastoma (GBM) is the most common and fatal type of primary brain tumor. Gliosarcoma (GSM) is a rarer and more aggressive variant of GBM that has recently been considered a potentially different disease. Current clinical treatment for both GBM and GSM includes maximal surgical resection followed by post-operative radiotherapy and concomitant and adjuvant chemotherapy. Despite recent advances in treating other solid tumors, treatment for GBM and GSM still remains palliative, with a very poor prognosis and a median survival rate of 12–15 months. Treatment failure is a result of a number of causes, including resistance to radiotherapy and chemotherapy. Recent research has applied the cancer stem cells theory of carcinogenesis to these tumors, suggesting the existence of a small subpopulation of glioma stem-like cells (GSCs) within these tumors. GSCs are thought to contribute to tumor progression, treatment resistance, and tumor recapitulation post-treatment and have become the focus of novel therapy strategies. Their isolation and investigation suggest that GSCs share critical signaling pathways with normal embryonic and somatic stem cells, but with distinct alterations. Research must focus on identifying these variations as they may present novel therapeutic targets. Targeting pluripotency transcription factors, SOX2, OCT4, and Nanog homeobox, demonstrates promising therapeutic potential that if applied in isolation or together with current treatments may improve overall survival, reduce tumor relapse, and achieve a cure for these patients.

## Introduction

Glioblastoma (GBM) is the most common and fatal type of primary brain tumor, with approximately 10,000 new adult cases per annum in the United States of America (1). It comprises 70% of all gliomas and is categorized as grade IV glioma. Gliosarcoma (GSM) is a rarer and more aggressive variant of GBM and comprises 1.8–2.8% of grade IV gliomas (2). Both GBM and GSM tumors grow quickly and are heterogeneous, composed of transformed glial cells. In addition, GSM contains a mesenchymal component within the glial component, which is thought to contribute to the increased heterogeneity and invasiveness of GSM. In line with these tumor characteristics, GSM is associated with a lower proportion of long-term survivors compared to the general pool of GBM patients (3, 4).

The absence of effective treatments for GBM and GSM, their invasiveness as well as their increasing incidence have sparked renewed interest in these tumors and their malignant characteristics (5, 6). A study between 2000 and 2008 presented an increasing incidence of grade IV gliomas from 3.22 to 3.96 cases per 100,000 people per year (6). Unfortunately, this is not the only concern about these tumors. Despite recent advances in treating solid tumors, treatments for grade IV gliomas remain palliative and do little to alter the very poor prognosis from this disease (7). Even the most aggressive surgical resections, although they debulk a large proportion of the tumor, always leave some residual that infiltrates surrounding brain tissue and recapitulates the tumor (5). GBM and GSM also display resistance to radiotherapy and chemotherapy, and often the recurrent tumors are more aggressive (4, 8). Prognosis for GBM and GSM patients is very poor, with a median survival of 12–15 months (9). These unsatisfactory outcomes stress the urgent need for the identification of novel therapeutic targets.

The recapitulation of the tumor after aggressive surgical resection and at times complete radiological responses following chemoradiotherapy suggests the presence and proliferation of cancer stem-like cells (CSCs) post-treatment (10). These have been identified in grade IV gliomas and are termed glioma stem-like cells (GSCs) (11). Recent studies are implicating pluripotency genes, normally expressed in self-renewing embryonic stem cells (ESCs) and in some types of plastic somatic cells (12, 13), in the pathology of GSCs, their origin, and persistency post-treatment. These genes may offer new promising therapeutic targets for this disease, with the aim to not only improve overall survival and achieve longer remission but also reduce the rate of tumor relapse, and potentially give rise to a cure for these devastating cancers.

## Glioblastoma and Its Subtypes

The Cancer Genome Atlas (TCGA) Research Network (2008) has generated an extensive catalog portraying the genomic characteristics of GBM. In addition, many groups have conducted high dimensional profiling studies in an attempt to understand the molecular mechanisms that drive tumorigenesis in GBM. These efforts have lead to the identification of several GBM tumor subtypes (14), of which the main subtypes are the classical, proneural, neural, and mesenchymal (14, 15).

The classical subtype displays mutations that create gene amplification and gene loss (14). In all classical subtypes screened, there was a loss of chromosome 10 and an amplification of chromosome 7, which is also seen in other GBM tumor subtypes. However, high levels of epidermal growth factor (*EGFR*) mutations are observed mainly in classical tumors, whereas they are rarely seen in other GBM subtypes (14). Classical tumors are also characterized by lack of tumor suppressor *TP53* mutation, whereas the majority of proneural tumors exhibit *TP53* mutation (15). In addition, the proneural subtype has two distinct features, namely alterations in platelet-derived growth factor receptor A (*PDGFRA*) and point mutation in *IDH1* (14). GBM tumors categorized as neural present expression of *NEFL, GABRA1, SYT1*, and *SLC12A5*, all of which are neuronal markers. On the other hand, mutations in neurofibromatosis type 1 (*NF1*) are characteristic of the mesenchymal category of GBM tumors (14). These different tumor subtypes may convey differing prognosis and response to treatments, which still remain unknown, yet reflect the heterogeneity of GBM (14).

Gliosarcoma, a morphological variant of GBM, also conveys a mesenchymal phenotype (3), associating GSM with the mesenchymal subtype of GBM (16). Expression of lineage-specific markers and morphological appearance suggests that the mesenchymal component is enriched for GSM allowing differentiation along multiple lineages including smooth and skeletal muscle (3, 17). These characteristics point toward a multilineage potential of GSCs in GSM. DNA profiling of GSM has revealed similar genetic profile to GBM, with mutations in tumor suppressors *TP53* and phosphatase and tensin homolog (*PTEN*). However, GSM is known to lack EGFR amplification, which is observed in some GBM tumors, especially the classical subtype (18). Reportedly, the pathology of GSM has been very little studied despite the worse prognosis of these patients (19).

## Current Glioblastoma Treatments

For many decades, post-operative radiotherapy was the only adjuvant therapy offered to patients diagnosed with grade IV gliomas (20). However, the addition of radiation treatment provided an overall survival benefit, but still with no long-term survivors (20). More recently, Stupp et al. (7) compared radiotherapy alone against radiotherapy with concomitant and adjuvant temozolomide (TMZ). The addition of TMZ, an oral alkylating agent, demonstrated anti-cancer activity and improved survival from 12.1 to 14.6 months. Most importantly, the proportion of 5-year survivors increased from around 2% to around 10% (9). In summary, the median survival after maximal surgical resection, combination of radiotherapy and concomitant chemotherapy, and also adjuvant chemotherapy is only 12–15 months, with a very small 10% of patients achieving long-term survival (9). Importantly, GSM patients are treated the same as the general GBM patients, although both molecular and phenotypic data suggest that GSM is an altogether different disease (17). However, due to the lack of additional encouraging data on other treatments, currently both GBM and GSM patients undergo maximal surgical resection followed by radiotherapy with concomitant and adjuvant chemotherapy with TMZ (9).

Temozolomide is not effective in all patients (21). TMZ is a lipophilic pro-drug able to penetrate the blood–brain barrier. Its metabolite, MTIC, has alkylating properties and deposits methyl groups to guanine bases of DNA. This creates nicks in the DNA strand causing cell cycle arrest between G2 and mitotic phase. Damage to DNA becomes overwhelming for the cell that leads to cellular apoptosis (22). The DNA repair enzyme O6-methylguanine-DNA methyltransferase (MGMT) is able to reverse the effects of TMZ by removing alkyl groups from the O6 position of guanine (23). Therefore, high levels of MGMT activity in cancers have been related to treatment failure of alkylating agents (23). Epigenetic silencing of MGMT through promoter methylation causes a loss in MGMT expression and prevents DNA repair (24). These findings suggested that MGMT could possibly be a predictive marker for chemotherapy with alkylating agents. Hegi et al. (21) validated this demonstrating that GBM patients with methylated MGMT promoter benefited from TMZ, whereas those with unmethylated MGMT promoters did not benefit from the treatment. Nevertheless, in the absence of other beneficial treatments in the unmethylated population and with evidence that a small proportion of patients with unmethylated MGMT do in fact benefit, all patients currently receive TMZ, and the presence of MGMT methylation does not currently determine treatment selection. However, there is an urgent need for new treatments for the unmethylated population.

Recurrent GBM can be different from the original tumor. TMZ is currently the standard treatment for newly diagnosed GBM, but there is no clear standard care for recurrent GBM. A characteristic of the recurrent tumors is high expression of vascular endothelial growth factor (VEGF), which has been associated with poor prognosis as it promotes angiogenesis and tumor growth (25). Phase II trials have been conducted in patients with recurrent GBM testing a combination of bevacizumab, a humanized immunoglobulin G1 monoclonal antibody for VEGF, and irinotecan, a topoisomerase 1 inhibitor (26, 27). The trials resulted in a median survival of approximately 7–9 months after treatment with bevacizumab and irinotecan (26, 27). Furthermore, immediate treatment with bevacizumab after surgery did not result in a survival benefit (28, 29). As there is no cure for grade IV gliomas, relapse occurs essentially in all patients. Recurrent GBM is also more likely to develop into its more aggressive variant GSM, which is characterized by shorter survival rate and at times can metastasize extracranially (8).

Collectively, it must be emphasized that current treatments for grade IV gliomas are only palliative and have shown limited survival benefits producing very poor prognosis and reinforcing the urgent need for the identification of novel therapeutic targets that will augment current therapies. A major explanation for the lack of effective treatments is the poor understanding of the cellular and molecular mechanisms governing tumor growth and recurrence. Focus of recent research has pointed toward CSCs.

## Cancer Stem-Like Cells

Cancer stem-like cells have recently become a main focus in cancer research. According to Clarke et al. (30), a CSC is a cancer cell able to self-renew and to differentiate into different cell lineages that contribute to the heterogeneity and the resulting complexity of tumors. The clonal evolution model suggests that self-renewal capabilities randomly occur (31). However, the CSCs hypothesis portrays a hierarchical structure in which stem-like cells are favored (32). CSCs are also known to be resistant to radiotherapy and chemotherapy and have the ability to remain quiescent (33); therefore, their persistence results in tumor redevelopment. Anti-cancer therapies may also switch the cellular hierarchy of the tumor toward CSCs (19, 34). This implies that CSCs could be the cause of the poor prognosis, treatment failure, and disease relapse associated with many solid tumors.

There has been intense discussion concerning the origin of CSCs. CSCs could derive from cancer cells that have been hierarchically downstream to give undifferentiated CSCs. Furthermore, cancer arises from the accumulation of mutations, thus CSCs may also originate from normal stem or progenitor cells. There is substantial evidence toward the connection between normal stem cells and cancer (34) in many tissues and organs. Researchers have isolated stem cells from the normal brain, and formed neurospheres in culture via the use of serum free media supplemented with cytokines (10). Each neurosphere is speculated to arise from a single stem cell (10) demonstrating their potential for self-renewal. Efforts have also been made to isolate CSCs from grade IV gliomas (11). GBM and GSM cells have been grown on non-adherent surfaces to form tumorspheres (35). Each sphere is thought to originate from a single CSC, similar to the normal neurospheres originating from a single neural stem cell (35). There are a number of examples illustrating the stem cell theory of carcinogenesis. Evidence has indicated that leukemia originates from leukemic stem-like cells (LSCs). (36). Furthermore, Al-Hajj et al. (37) demonstrated that a small minority of cells within breast cancer express CD44 and CD24 surface markers, which distinguish and isolate tumor-initiating cells from non-tumorigenic cells (37). CSCs have also been found in human ovarian cancers (38). The identification and isolation of CSCs from solid human brain tumors (10), leukemia (36), ovarian cancer (38), and breast cancer (37) have been achieved and provide a unique opportunity for exploring the tumor-initiating and -maintaining abilities of CSCs.

## Cancer Stem-Like Cells in Brain Tumors

**Figure 1:**
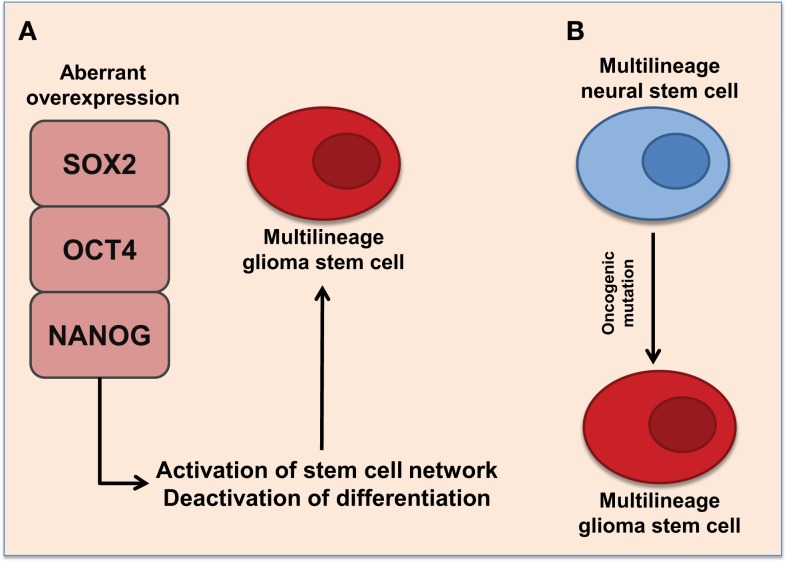
**Proposed representations of the development of glioma stem cells**. **(A)** Aberrant overexpression of pluripotent transcription factors, SOX2, OCT4, and NANOG, promote multilineage potential in glioma stem cells. Aberrant overexpression of pluripotent transcription factors activates stem cell networks while deactivating differentiation pathways. This promotes the formation of self-renewing glioma stem cells with multilineage potential. **(B)** Multilineage neural stem cells express SOX2, OCT4, and NANOG. Oncogenic mutations may cause aberrant expression of pluripotent transcription factors resulting to multilineage glioma stem cells.

The brain contains neural stem/progenitor cells, and the possibility has been suggested that aggressive gliomas occur by mutations of these cells (Figure 1B). Previous studies have supported this by showing that GBM contains tumor cells comparable in some ways to normal neural stem cells (39). Normal neural stem cells express CD133 (also called Prominin 1, PROM1) (40), a cell surface glycoprotein, the function of which is still poorly understood. CSCs from human gliomas may also express CD133 (40). Further investigations showed that within a tumor, there is a population of cells that are either CD133+ or CD133−, with the former being able to recapitulate the tumor (10). The link between Phillips et al. (15) and Verhaak et al. (14) molecular subtype of GBM and GSCs is not established, but analysis has presented that the mesenchymal and neural subtypes demonstrates strong signatures of CD133 expression (41). *In vitro* and *in vivo* studies conveyed that GSCs from mesenchymal subtypes were more aggressive, invasive, and more resistant to treatment than classical and neural subtypes (42), suggestive of a correlation with CD133 expression. However, later investigations showed that subpopulations within the CD133− population could also generate tumors (43). Interestingly, neurospheres and serum cultures of GSM lacked CD133 expression (3), presenting the controversy of the CSC theory as no markers for CSCs have been established (19). Collectively, these data emphasize the existence of a cellular hierarchy including plastic, undifferentiated CSCs in solid brain tumors (10, 11). Both CD133+ and CD133− cell populations have been shown to lack expression of neural differentiation markers suggesting mutagenic transformation from neural stem cells and derivation of cells of multiple differentiation states within a tumor (10).

Campos et al. (44) applied a neural colony-forming assay to GBM cell lines to examine self-renewal and tumor-forming capacities, and established that GBM cultures displayed low and high clonogenic subpopulations that contribute to aberrant tumor growth and tumor recurrence. Culturing of GBM cells under conditions permissive for stem cell proliferation generated tumorspheres with self-renewing capacities, which is a shared property of neural stem cells (11, 44). Again, this hints at the existence of CSCs in gliomas. These spheres could produce daughter cells that displayed the phenotypes present in GBM (11). Similarly, GSM cells placed in neurosphere promoting media were able to form tumorspheres, which were transplanted into mice and consequently generated large necrotic tumors (3). Reiterating, the proposed existence of CSCs in glioma, but very little research has explored the GSCs population in GSM.

Normal neural stem cells are known to exist within several brain niches, such as the vascular niche (45). Evidence also implicates that CSCs within brain tumors are harbored and maintained within vascular niches (46). Most brain tumors are highly vascularized, hence there has been speculation about a close relationship between CSCs and blood vessels. Calabrese et al. (46) demonstrated that vascular endothelial cells were able to maintain the stem-like properties and tumorigenicity of brain tumor cells in immunocompromised mice. Vascular endothelial cells also release nitric oxide, which has been shown to promote self-renewal and maintain CSCs through activation of the Notch signaling pathway (47). The stimulation of Notch signaling via the vascular niche could possibly aid resistance to radiotherapy and chemotherapy (48). Thus, the vascular niche could be a target for novel therapies.

**Figure 2:**
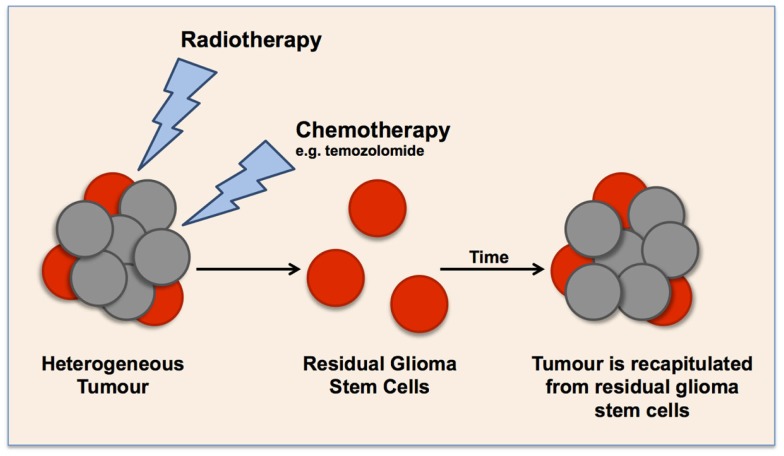
**Schematic representation of the cancer stem cell theory in glioma tumors, illustrating the effect of current clinical treatments on enriching glioma stem cell populations**. Current radiotherapy and chemotherapies target highly proliferative cells, leaving a small population of quiescent cells that over time cause the recapitulation of the tumor.

As mentioned above and following the CSC theory of carcinogenesis, this subpopulation of cancer cells has the capacity to self-renew aiding to the maintenance of the tumor (39). However, CSCs are quiescent and are cycling slowly (33, 49). It is this feature as well as their ability to asymmetrically divide that is thought to contribute to their chemo-resistance and the recapitulation of the tumor post-treatment, respectively. More specifically, GBM have shown to harbor quiescent cells that are non-apoptotic (44), resulting in their survival after chemotherapy and radiotherapy, both of which target cycling, highly proliferative cells (33, 49). In turn, the ability of CSCs to divide asymmetrically and give rise to more differentiated daughter cells that are more proliferative is thought to facilitate the regrowth of the tumor post-treatment, and it also contributes to the heterogeneous phenotype of the tumor (Figure 2) (30, 39). This theory has been implicated in relation to GBM as quiescent cells triggered tumor regrowth after TMZ treatment (50). These characteristics perfectly describe the malignant nature of grade IV gliomas, and portray that these cells contribute to the aggressiveness, tumor progression, and recurrence. Therefore, to achieve long-lasting remission or a cure for GBM and GSM, we need to target GSCs as well as the highly proliferative cells. Targeting GSCs should augment current clinical treatments.

## Treatment Resistance of Glioma Stem-Like Cells

The common cause for treatment failure in many malignancies, including grade IV gliomas, is tumor resistance to radiotherapy and chemotherapy (21, 51, 52). Collective data have established that CSCs, such as GSCs, are more resistant to conventional radiotherapy and chemotherapy than non-CSCs (30). Glioma xenografts subjected to radiation were enriched for cells expressing CD133, suggesting that radiotherapy enhances the GSCs population (51). GSCs possess the ability to activate several checkpoint proteins, such as ATM, Rad17, Chk2, and Chk1, in response to DNA damage induced by radiation (51). Hence, GSCs are able to efficiently repair damaged DNA, allowing better recovery than non-stem tumor cells. Low molecular weight inhibitors against Chk1 and Chk2 kinases eliminated radio-resistance and sensitized GSCs to radiotherapy, but the inhibition of such DNA damage checkpoints in normal stem cells may lead to oncogenesis and is therefore prohibitive (51). Another pathway that demonstrates radio-resistance is the Notch signaling pathway. Notch signaling promotes self-renewal and its suppression by γ-secretase inhibitor or Notch shRNA promotes sensitivity of GSCs to radiation (52).

Beside radio-resistance, GSCs have also shown resistance to clinically used chemotherapies. As described above, chemo-resistance to TMZ occurs in patients with high expression of MGMT, a DNA repair gene (21). GSCs also highly express BCPR1, a drug resistance gene (53). This further contributes to chemo-resistance to TMZ (53).

Treatment resistance can also be explained using the CSCs hypothesis. As described above, the CSC hypothesis presents a hierarchical structure, which favors stem-like cells, such as GSCs (32). Radiotherapy and chemotherapies could enhance this phenotype by switching the cellular hierarchy toward GSCs (19, 34). It is known that radiotherapy and chemotherapy target highly proliferative cells and are thus ineffective on quiescent, slow-cycling GSCs. Moore and Lyle (49) demonstrated that cell lines with low clonogenic capacity failed to grow detectable tumors *in vivo* but highly clonogenic cell lines quickly produced large tumors. Thus, current clinical treatments enrich the GSCs subpopulation, which overtime recapitulates the tumor due to its self-renewal properties (33, 44, 49).

Current knowledge has failed to identify a novel therapeutic target for grade IV gliomas that will produce better prognosis, longer-lasting remission, or a possibility of a cure without inflicting harm to normal brain cells and normal neural stem cells.

## Targeting Glioma Stem-Like Cells

One search avenue for effective therapeutic targets for gliomas has focused on exploiting the self-renewal and other critical pathways in GSCs, while causing minimal toxicity to normal cells. The CSC hypothesis proposes that research must identify critical pathways controlling maintenance of CSCs that do not overlap with those needed by normal cells (54). Direct targeting of GSCs may improve the efficacy of conventional radiotherapy and chemotherapy through eliminating residual resistant stem cells. In this context, signaling pathways and transcription factors overexpressed or specifically activated in GSCs have been investigated in hope for a novel therapeutic target.

Notch signaling is one of the many pathways examined. There are four different members of the Notch protein family (1–4), all of which are transmembrane receptors that mediate short-range cellular communication (55). The Notch signaling pathway generally acts to promote self-renewal and repress cellular differentiation, and it is therefore essential for the maintenance of stem and progenitor cells (55). As mentioned above, inhibiting Notch signaling through γ-secretase inhibitor or Notch shRNA reduced radio-resistance (52). Blockade of Notch signaling in GSCs has also diminished the capability to form tumorspheres (56). Blocking the Notch signaling pathway evidently may be a good target for GSCs; however, *in vivo* studies show that cells pretreated with γ-secretase inhibitors still developed large xenografts (56). Another signaling pathway overexpressed in grade IV gliomas is the signal transducer and activator of transcription 3 (STAT3) (57). STAT3 is involved in many cellular activities, such as cell growth, division, and apoptosis (58). Abnormal expression of STAT3 in GSCs promotes cell growth and contributes to immunomodulation (57). Inhibitors that induce genetic knockdown of STAT3 interrupt proliferation and maintenance of GSCs as well as decreased tumorigenic capabilities *in vivo* (57). However, STAT3 is also vital for the maintenance of normal stem cells and is a critical component of normal immune responses; therefore, targeting STAT3 will not be specific to tumor cells and may cause major side effects (57).

It has also been shown that GBM cells have low expression of miR-145, which is associated with poor patient outcome (59). The knockdown of miR-145 expression in GSCs leads to increased cell proliferation, invasion, and migration, whereas upregulation causes the opposite effects (59). Further investigations of the mechanisms behind loss of miR-145 expression in GBM revealed a negative correlation with ABCG2 (60). ABCG2 is an ATP-binding cassette transporter protein known to be overexpressed in GSCs (61). Dual-luciferase reporter gene assays showed that ABCG2 is a target for miR-145 (60). Knockdown of ABCG2 by small interfering RNA reduced cell migration and invasion (60). This reveals the importance of miR-145 in preventing tumor progression and potentially proposes that upregulation of miR-145 or downregulation of ABCG2 could be possible novel targets for grade IV gliomas.

As mentioned above, there is some controversy concerning the CSC theory as cell marker or identifiers for GSCs have not been established, which limits the isolation and study of GSCs (19). The search for novel therapeutic targets may also identify possible novel markers or identifiers for the GSCs population, aiding in the isolation and understanding of the GSCs population.

## Function of Pluripotency Genes in Normal Stem Cells and in Glioma

Glioma stem-like cells share important characteristics with normal stem cells (12, 39), including key stem cell transcription factors that are involved in cell maintenance (Figure 1). Some of these transcription factors are sex determining region Y-Box (SOX2), octamer-binding transcription factor 4 (OCT4), and Nanog homeobox (NANOG), which are critical components in maintaining pluripotency in ESCs and somatic stem cells (12, 19, 62, 63). SOX2, OCT4, and NANOG are known to be highly expressed in subpopulations of GSCs, maintaining self-renewal and cellular proliferation (64). They are also thought to contribute to the multilineage potential and heterogeneity of GSCs (64). SOX2 expression is very minimal in the adult brain and is only limited to stem cells and progenitor cells (65), thus emphasizing the possibility of SOX2, as a potential therapeutic target for grade IV gliomas. The expression of OCT4 and NANOG in normal brain tissue is still unclear.

**Figure 3:**
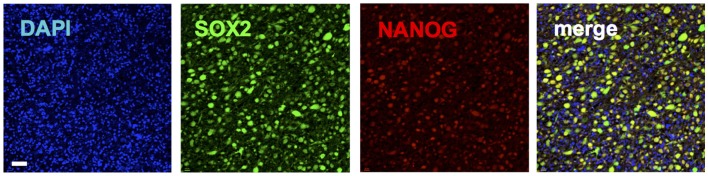
**Immunohistochemical staining of a glioblastoma patient tumor sample depicting co-expression of pluripotency genes SOX2 (green) (20) and NANOG (red)**. Blue shows nuclear staining (DAPI).

*SOX2* is a gene that encodes a transcription factor made of 317 amino acids (62). *SOX2* contains a high mobility group (HMG) DNA-binding domain (62). OCT4 is a member of the Pit–Oct–Unc (66) transcription family known to interact with other transcription factors to activate and repress genes (67). OCT4 heterodimerizes to SOX2 and synergistically alters the expression of several genes in ESCs (62). NANOG is a homeobox protein, which also cooperates with SOX2 and OCT4 (Figure 3) in the regulation of genes, vital at the early development of ESCs (68). SOX2, OCT4, and NANOG co-occupy the promoter regions of at least 353 genes (62). Among these are genes encoding key signaling pathways that control pluripotency and self-renewal (62). At the same time, they repress genes that promote differentiation (62, 68). These transcription factors are also involved in an autoregulatory loop controlling their own genes (62). In addition to ESCs, SOX2 is expressed in neural stem cells and prevents their differentiation into neurons (69). It is also expressed in other adult stem cells, such as those of the breast, particularly during pregnancy and lactation (63). SOX2 is also expressed in stem/progenitor populations in the liver, pancreas, and stomach, which are endodermal organs (70).

A mouse model revealed that SOX2 is required for the maintenance of CSCs in high-grade oligodendroglioma (71). Mice transplanted with SOX2-depleted cells remained tumor-free, whereas control animals produced lethal tumors. This highlights a potentially important role for SOX2 in brain neoplasm tumorigenicity. In the same study, a subgroup of wild type mice was vaccinated with a SOX2 peptide. This SOX2 immunotherapy increased survival rates and the combination of peptide with TMZ-doubled survival time (71).

Guo et al. (13) used qRT-PCR and western blotting to demonstrate SOX2 expression in gliomas. Western blot analysis demonstrated that grade IV gliomas had greater SOX2 mRNA expression than grade II gliomas (13). The function of SOX2 has also been specifically characterized in GBM tumor-initiating cells (64). The silencing of SOX2 in human GBM cells transplanted in immunodeficient mice ceased cell proliferation and resulted in loss of tumorigenicity (64). Gangemi et al. (64) also confirmed that the observed effect was due to SOX2 knockdown.

In addition to SOX2, expression of OCT4, a known partner of SOX2 (62), has also been reported in human gliomas, with higher grade gliomas showing significantly greater mRNA expression than low-grade gliomas (72). Immunostaining illustrated the localization of OCT4 in the nucleus of tumor cells, with higher grade gliomas staining more intensely than low-grade gliomas (72). Interestingly, the expression of OCT4 has not been characterized in grade IV gliomas.

Nanog homeobox is another transcription factor playing a crucial role in the self-renewal and differentiation of ESCs; therefore, it is no surprise that NANOG expression has been detected in human gliomas (73). Furthermore, a positive correlation between NANOG expression and pathological grade was observed (72, 73). Grade III and IV glioma tissues presented strong expression and localization of NANOG in the nuclei of glioma cells, whereas lower grade gliomas displayed low to moderate expression of this gene (73).

Current research demonstrates a positive correlation between the expression of SOX2, OCT4, and NANOG and the pathological grade of gliomas (73). The aberrant expression of SOX2, OCT4, and NANOG may promote self-renewal as well as multilineage potential within GSCs (Figure 1A). However, there is a possibility that these transcription factors may exhibit a distinct role in individual tumors, and the variation among different GBM subtypes and in GSM is as yet unexplored. It can also be hypothesized that the increased aggressiveness of recurrent GBM and the conversion to GSM that is sometimes seen is due to the enhancement of the GSC phenotype post-treatment. Indeed, it has been recently showed that expression of OCT4 and NANOG increased after anti-EGFR therapy in GBM (74). Thus, further investigation is warranted to characterize the expression of SOX2, OCT4, and NANOG in grade IV gliomas and examine potential associations with patient outcome and tumor aggressiveness. Moreover, investigations examining the effects of current clinical treatments on the expression of these genes in GBM and GSM may elucidate the mechanisms of tumor recurrence and treatment failure.

## SOX2, OCT4, and NANOG Target Therapies in Other Tumors

SOX2 studies in ovarian epithelial lesions revealed that SOX2 expression increased with malignant potential from benign, borderline to malignant ovarian tumors (75). The expression of SOX2 within serous ovarian cancer cells induced properties of CSCs, such as increased expression of CSC markers, the ability to form tumorspheres, their tumor-initiating capacity, and the enhanced ability to resist conventional chemotherapies (76), characteristics similar to those displayed by gliomas. SOX2 has been shown to be a major player in the tumorigenicity of breast cancers. The overexpression of SOX2 has been demonstrated in 43% of basal cell-like triple negative breast carcinoma and 28% of all invasive breast carcinoma (77). Stolzenburg et al. (77) engineered zinc-finger-based artificial transcription factors that selectively suppressed SOX2 gene expression. This technology was tested in breast cancer cell lines and resulted in a significant 74–94% downregulation of SOX2 mRNA expression compared to an empty vector control. Human breast tumor xenografts grown in mice were also treated with these artificial transcription factors and produced a significant reduction in tumor size compared to non-transduced tumors (77).

Similarly, OCT4 has also been detected in ovarian cancer cell lines and tumor patient samples, advancing with tumor grade (78). The downregulation of OCT4 via RNA interference caused a 60% reduction in cell viability (78). The same trend was observed with NANOG mRNA expression, as ovarian cancers expressed significantly higher levels than benign cystadenomas (79). NANOG expression also defined patient outcomes and poor prognosis, while its knockdown reduced cell proliferation, migration, and invasion (79).

These studies reinforce the importance of targeting key regulatory transcription factors to reduce their proliferative potential and facilitate successful treatment of malignancies. It also opens up investigations of the use of such and/or similar technologies in other types of cancers like GBM and GSM that express these transcription factors. With such treatments and brain tumors, the blood–brain barrier can implicate a challenge; however, such treatments be administered during surgical resection. In relation to GBM and GSM, similar techniques for the administration of carmustine, another chemotherapy drug, can be used to give these novel therapies.

## Conclusion and Outlook

Grade IV gliomas are a difficult cancer to treat and remain incurable. Treatment for grade IV gliomas has not seen any major recent therapeutic advances, with the most exciting advances providing very minor improvements in survival. Recent research has followed the CSC theory of carcinogenesis, implicating that grade IV gliomas contain GSCs. This small population of GSCs has shown powerful capabilities of invasion, therapeutic resistance, and tumor recapitulation post-treatment. Therefore, the exploitation of GSCs may elude the mechanisms governing treatment resistance and tumor recurrence seen in all grade IV glioma patients.

Cancer cures need to eliminate all tumor cells. Targeting GSCs within grade IV gliomas may help improve the poor prognosis and provide the possibility of a cure. However, GSCs share critical signaling pathways as normal neural stem cells thus targeting GSCs proves to be a difficult task. We have discussed targets and pathways, such as Notch and STAT3 (55, 57), which have shown promising potential but demonstrated no clinical application as they are vital for the function of normal stem cells. Future research should focus on identifying molecular regulators and signaling pathways that are exclusively unique to GSCs. The amplified expression levels of SOX2, OCT4, and NANOG transcription factors in grade IV gliomas implicate a possible clinical intervention. SOX2 has previously shown great potential. A breast cancer study used zinc-finger-based artificial transcription factors to downregulate SOX2 expression, which consequently lead to reduction in tumor size (77). Breast cancer cells lines have also been treated with another similar technology known as synthetic interference peptides (80). Synthetic interference peptides are a new technology that specifically targets transcription factors to inhibit their aberrant function. These therapeutic strategies can be applied to GBM and GSM; however, further investigation is required to determine the clinical relevance of these laboratory techniques, and the effect of current clinical treatments on GSCs. These novel therapies should also augment current clinical treatment to eliminate the tumor as a whole. GBM is a heterogeneous disease with tumors varying molecularly and genetically between patients, suggesting the use of several targeted therapies. Current research has also suggested that GSM is a completely different disease to GBM. Thus, further investigation is required to clearly define the genetic and phenotypic characteristics of GSM in order to develop patient-specific and -effective treatments.

## Conflict of Interest Statement

The authors declare that the research was conducted in the absence of any commercial or financial relationships that could be construed as a potential conflict of interest.
